# Real-World Retrospective Study of Clinical and Economic Outcomes Among Patients with Locally Advanced or Metastatic Urothelial Carcinoma Treated with First-Line Systemic Anti-Cancer Therapies in the United States: Results from the IMPACT UC-III Study

**DOI:** 10.3390/curroncol32070384

**Published:** 2025-07-02

**Authors:** Helen H. Moon, Chiemeka Ike, Ruth W. Dixon, Christopher L. Crowe, Malvika Venkataraman, Valerie Morris, Mairead Kearney, Ivy Tonnu-Mihara, John Barron

**Affiliations:** 1 Kaiser Permanente Southern California, Riverside, CA 92505, USA; helen.h.moon@kp.org; 2 EMD Serono, Inc., Boston, MA 02210, USA; chiemeka.ike@emdserono.com (C.I.); valerie.morris@emdserono.com (V.M.); 3 Carelon Research, Wilmington, DE 19801, USA; christopher.crowe@carelon.com (C.L.C.); malvika.venkataraman@carelon.com (M.V.); qtonnu@yahoo.com (I.T.-M.); john.barron@carelon.com (J.B.); 4 Merck Healthcare KGaA, 64293 Darmstadt, Germany; mairead.kearney@merckgroup.com

**Keywords:** bladder cancer, first-line treatment, healthcare costs, immune checkpoint inhibitors, metastatic urothelial carcinoma, outcome assessment, real world, systemic therapy, treatment patterns

## Abstract

This retrospective cohort study evaluated characteristics, treatment patterns, and clinical outcomes in adults with locally advanced/metastatic urothelial carcinoma (la/mUC) receiving first-line (1L) systemic treatment with or without avelumab 1L maintenance (1LM) between January 2020 and July 2023. The index date was the first date with a claim for 1L systemic therapy after a la/mUC diagnosis. Patients with continuous health plan enrollment for ≥6 months before and ≥1 month after the index date were identified from Carelon Research’s Healthcare Integrated Research Database. Of 2820 patients receiving 1L treatment, 37.0% received platinum-based chemotherapy (PBC); 39.0%, immuno-oncology (IO) monotherapy; and 24.0%, other therapies. Renal disease and other comorbidities influenced 1L regimen choice. Healthcare resource utilization (HCRU) and costs were reported for patients receiving second-line (2L) treatment. HCRU was high in 32.8% of patients (926 of 2820) who received 2L treatment. Median all-cause direct medical costs per patient per month were USD 15,859, USD 19,781, USD 11,346, and USD 9516 for 1L PBC, 1L PBC + avelumab 1LM, 1L IO monotherapy, and 1L other therapies, respectively. Most direct healthcare costs were attributed to all-cause outpatient visits.

## Simple Summary

This study evaluated real-world outcomes of first-line treatments for advanced or metastatic bladder cancer in the US from 2020 to 2023, using de-identified health records from 2820 patients. Treatments included chemotherapy (37%), immunotherapy (39%), and other therapies (24%). Socioeconomic factors and conditions like kidney disease influenced treatment choices. Outpatient visits emerged as a major cost driver, with costs varying between treatment types. Findings emphasize the importance of patient characteristics in treatment decisions and the economic impact of therapies. This study offers insights that can influence future research, policy, and real-world applications. Policymakers may use these findings to create guidelines ensuring equitable access to effective cancer treatments. Clinicians can improve decision making by considering individual patient characteristics like age and socio-economic status. Insurance providers might adjust coverage plans to support beneficial therapies in the first line, emphasizing cost-effective management for advanced or metastatic bladder cancer care.

## 1. Introduction

Urinary bladder cancer is the sixth most prevalent cancer overall in the United States (US) and predominantly affects older men [1]. There are several histological types of bladder cancer, with the urothelial carcinoma (UC) subtype accounting for over 90% of all cases [1,2]. Understanding the histologic disease subtype and disease extent of UC is important to determine the appropriate treatment approach [1]. Approximately one-quarter of patients with UC present with locally advanced/metastatic urothelial carcinoma (la/mUC), an aggressive disease with a 5-year survival rate of <10% [3,4].

Bladder cancer treatment costs in the US are high, especially for patients with la/mUC, and increase with progression through lines of therapy (LOTs). The annual average cost per newly diagnosed patient with muscle-invasive bladder cancer was found to range from USD 35,545 to USD 169,553 for those with metastatic disease in 2021 [5].

Treatment modalities for patients with la/mUC include chemotherapy, targeted therapy, immune checkpoint inhibitors, antibody–drug conjugates (ADC), or a combination of therapies [1]. The presence or absence of comorbidities is a factor in recommendations for systemic anticancer therapy [1]. Platinum-based chemotherapy (PBC) has historically been the standard first-line (1L) systemic therapy for patients with la/mUC; however, age-associated renal function and performance status impairment may affect some patients’ eligibility for cisplatin treatment [1,6,7]. Over the past few years, treatment options for patients with la/mUC have expanded rapidly, beginning with the introduction of immune checkpoint inhibitors, including pembrolizumab, nivolumab, and avelumab [8,9,10,11,12]. For patients with la/mUC without disease progression on 1L PBC, avelumab 1L maintenance (1LM) is a standard of care based on the results of the JAVELIN Bladder 100 clinical trial [8,9,13,14].

More recently approved 1L regimens incorporated into clinical guidelines include enfortumab vedotin (EV) in combination with pembrolizumab, regardless of cisplatin eligibility, and nivolumab, gemcitabine, and cisplatin in cisplatin-eligible patients [1,15]. Immuno-oncology (IO) monotherapy with an immune checkpoint inhibitor is considered in patients ineligible to receive PBC [1]. Other regimens that can be used for cisplatin-ineligible patients include gemcitabine + carboplatin (followed by avelumab 1LM); gemcitabine alone or with paclitaxel; or atezolizumab for patients whose tumors express programmed death-ligand 1 (PD-L1). The split-dose administration of cisplatin is also an option in patients who would otherwise be deemed cisplatin-ineligible (due to advanced age, renal dysfunction, or other comorbidities) [1,16].

The choice of second-line (2L) systemic therapy depends on whether the 1L therapy was PBC, another chemotherapy, or an IO [1]. Current 2L treatment options include IO monotherapy, erdafitinib (for patients harboring *FGFR3* alterations), ADC monotherapy with EV, sacituzumab govitecan, or trastuzumab deruxtecan, and various chemotherapy regimens [1,17,18,19]; however, the accelerated approval of sacituzumab govitecan for la/mUC in the US has been voluntarily withdrawn by the manufacturer [20].

Treatment sequencing incorporating newly approved agents for la/mUC is not well characterized, although the treatment landscape is expected to evolve in the coming years as therapies move to earlier disease stages. Therefore, understanding patient characteristics, treatment patterns, clinical outcomes, healthcare resource utilization (HCRU), and total cost of care is crucial for effective disease management [1,5,21]. The present US retrospective study evaluated the real-world use of 1L systemic therapies, including avelumab 1LM and other newly approved therapies, and their impact on clinical and economic outcomes in patients with la/mUC using a US-based integrated dataset.

## 2. Materials and Methods

### 2.1. Study Design and Time Periods

This US-based study was a noninterventional, retrospective cohort study of patients with la/mUC aged ≥18 years who initiated 1L systemic treatment. The index date was the first date with a claim for 1L systemic therapy after a la/mUC diagnosis. The patient identification period was 1 January 2020 to 31 July 2023. The study period was 1 July 2019 to 31 August 2023, allowing for a baseline period of ≥6 months and ≥1 month of follow-up after the index treatment date (Figure S1).

### 2.2. Data Sources

Patients were identified from the Carelon Research Healthcare Integrated Research Database (HIRD^®^), including claims data, social determinants of health (SDoH) data, mortality data, and clinical data from the health plan’s Cancer Care Quality Program (CCQP).

HIRD^®^ data were obtained from commercial and Medicare health plans across the US representing members in each of the 50 states. The 2020 HIRD^®^ population (commercial and Medicare) is representative of the 2020 US Census population in terms of sex (male/female; overlap index = 99.2%), age (5-year age categories; overlap index = 92.0%), and geographic region of residence (Northeast/Midwest/South/West; overlap index = 94.8%) [22,23].

SDoH includes key metrics at both the individual (race/ethnicity and urbanicity) and area levels (economic stability, education, neighborhood, and built environment). The individual-level race or ethnicity variable was derived from a combination of enrollment data, self-attestations, electronic medical records, and algorithm-derived imputations (using name and geography). SDoH indicators are integrated in the HIRD^®^ using the most recent publicly available, nonproprietary data; the source files are from the American Community Survey, the National Center for Health Statistics (NCHS), and the Food Access Research Atlas [22,24,25,26]. The socioeconomic status (SES) index is a composite measure of multiple SDoH variables (e.g., education attainment, median family income, poverty rate, unemployment rate, crowding, and median home value). Compared with the US general population and using all US Census block groups as the reference basis for calculation, a score of 4 indicates the patient is in the top 25% of SES, and a score of 1 indicates the patient is in the bottom 25% of SES [24,25,26].

Mortality was identified in the HIRD^®^ through a combination of sources, including inpatient discharge status from claims, reason for insurance disenrollment, linking to obituary data, and linking to the Death Master File (DMF) from the Social Security Administration.

CCQP data have been available since July 2014 for a subgroup of patients whose healthcare providers submitted treatment pre-certification requests. These data include important clinical information, such as cancer type, staging, biomarkers, pathology or histology, line of treatment, weight and height, and performance status. Additional information on the data sources is provided in the Supplementary Data S1 and S2.

The data were used in full compliance with the relevant provisions of the Health Insurance Portability and Accountability Act of 1996. The study was conducted under the research provisions of Privacy Rule 45 Code of Federal Regulations 164.514(e) and was exempt from institutional review board evaluation.

### 2.3. Patient Population

**Figure 1:**
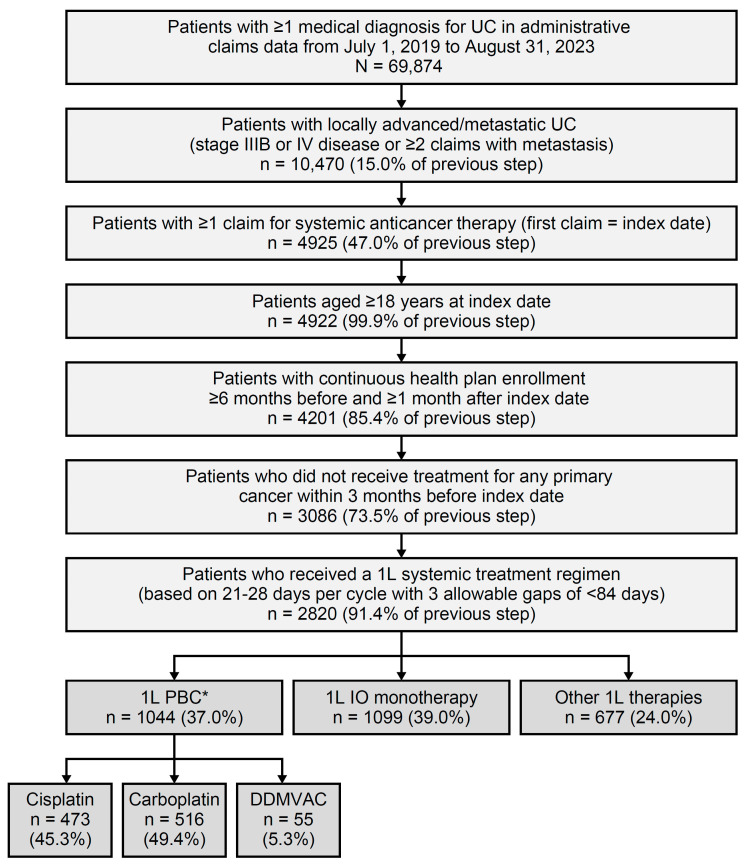
Patient selection and attrition. 1L, first line; DDMVAC, dose-dense methotrexate, vinblastine, doxorubicin, and cisplatin; IO, immuno-oncology; PBC, platinum-based chemotherapy; UC, urothelial carcinoma. * Among patients with 1L PBC, 15.0% (157 of 1044) initiated avelumab as maintenance therapy within 90 days of completing 1L PBC.

Patient selection is shown in Figure 1, alongside attrition. Patients were included if they were aged ≥18 years as of the index date, had ≥1 medical claim with a diagnosis code for UC in any position, and had received either a diagnosis of la/mUC—defined as either stage IIIB or IV bladder cancer in CCQP data—or had ≥2 medical claims with a secondary neoplasm diagnosis during the study period. Patients were also required to have ≥1 claim for systemic anticancer therapy after la/mUC diagnosis. The index date was the first date when systemic therapy was received. In addition, patients were required to have continuous health plan enrollment with medical benefits for ≥6 months before and ≥1 month after the index date. Exclusion criteria included the use of treatment(s) for any primary cancer during the 3 months pre-index and any participation in clinical trials during the study period. Patient consent was waived for this study because it retrospectively analyzed an anonymized dataset and did not directly involve human subjects.

### 2.4. LOTs Algorithm

Regimens for each LOT were determined based on all systemic treatments received within 21 to 28 days, which was considered the standard chemotherapy and IO cycle for patients with la/mUC. 1L treatment was defined as the first regimen of the first cycle that began on or after the date of la/mUC diagnosis. The end of 1L treatment occurred at treatment switch, treatment discontinuation, end of study period, end of continuous enrollment, or death, whichever came first. A new and next LOT was considered when the gap between regimen cycles was ≥84 days. Avelumab as 1LM was identified when claims for avelumab were seen after 30 June 2020 (i.e., date of US Food and Drug Administration [FDA] approval) [27] and within 90 days of the last day of 1L PBC administration.

### 2.5. Treatment Categories

Patients with la/mUC were categorized into the following cohorts based on the 1L systemic treatment received: 1L PBC, 1L IO monotherapy, and 1L other therapies, including ADC, targeted therapies, and non-platinum chemotherapies. Patients receiving 1L PBC were further stratified into the following 3 mutually exclusive cohorts: carboplatin-based chemotherapy, cisplatin-based chemotherapy, and dose-dense methotrexate, vinblastine, doxorubicin, and cisplatin (DDMVAC). An additional cohort of patients was included who initiated avelumab 1LM within 90 days of completing 1L PBC (1L PBC + avelumab 1LM).

### 2.6. Clinical Outcome Measures

Clinical outcomes, including time to next treatment (TTNT) and overall survival (OS), were evaluated during the available follow-up time. TTNT was defined as the time from 1L treatment initiation to 2L treatment initiation. OS was defined as the time from 1L treatment initiation to the date of death, accounting for the end of continuous enrollment or the study period. Treatment-free interval, defined as the time between 1L treatment cessation and avelumab 1LM initiation, was reported for the 1L PBC + avelumab 1LM cohort [28].

### 2.7. HCRU and Costs

All-cause and UC-related HCRU and healthcare costs were calculated for all patients with la/mUC receiving both 1L and 2L treatment and presented for the number and percentage of patients with ≥1 visit. All-cause and UC-related inpatient hospitalizations, length of inpatient stay (days), emergency room (ER) visits, and all types of outpatient visits were reported. Healthcare costs were adjusted to 2023 US dollars using the Consumer Price Index from the US Bureau of Labor Statistics. The number of visits and healthcare costs are reported as per patient per month (PPPM), to account for the variable length of follow-up.

### 2.8. Statistical Analysis

Sample selection, variable creation, data manipulation, and statistical analysis were performed using the Instant Health Data^®^ Analytics platform (Panalgo, Boston, MA, USA), R version 4.0.2 (R Foundation for Statistical Computing, Vienna, Austria), and Statistical Analysis Software Enterprise Guide, version 9.3 (SAS Institute Inc., Cary, NC, USA).

Patient demographics, baseline characteristics, cancer treatment history, and treatment patterns were described using univariate statistics including frequencies, percentages, mean, median, standard deviation (SD), and interquartile range (IQR). OS was analyzed using Kaplan–Meier methodology. The number of patients at risk and number of patients who died were reported throughout follow-up and at prespecified landmarks of 1, 2, and 3 years.

HCRU and costs were reported as continuous variables in the form of mean, SD, median, and IQR. Statistical differences were simultaneously assessed for 1L PBC versus 1L IO monotherapy versus 1L other therapy using 1-way analysis of variance or Kruskal–Wallis test for continuous variables, while chi-squared (χ^2^) or Fisher’s exact tests were used for categorical variables.

No comparison or analysis for statistical differences was completed across sub-cohorts. No data were imputed, and variables with undocumented values were categorized as missing/unknown.

## 3. Results

### 3.1. Patient Attrition

Of 2820 patients diagnosed with la/mUC and initiating 1L systemic treatment, 37.0% (1044 of 2820) received 1L PBC, 39.0% (1099 of 2820) received 1L IO monotherapy, and 24.0% (677 of 2820) received 1L other therapies (Figure 1).

Among the patients receiving 1L PBC, 45.3% (473 of 1044) received cisplatin-based treatment; 49.4% (516 of 1044), carboplatin-based treatment; and 5.3% (55 of 1044), DDMVAC. The cisplatin-based PBC and DDMVAC sub-cohorts were mutually exclusive (Figure 1; see Tables S1 and S2 for results of the 1L PBC sub-cohorts).

Among patients treated with 1L PBC during follow-up, 15.0% (157 of 1044) initiated avelumab 1LM. The annual uptake of avelumab as 1LM was 10.8% in June–December 2020, 29.9% in 2021, 34.4% in 2022, and 24.8% in January–August 2023; 38.9% (61 of 157 patients) were still receiving avelumab 1LM at the end of the study period.

### 3.2. Patient Demographics and SDoH Characteristics

Patient demographics and SDoH characteristics are presented in Table 1. Overall, 69.2% (1951 of 2820) were male, and 73.0% (2058 of 2820) were White non-Hispanic or Latino. Patients in the 1L IO cohort had a median age of 76 years (IQR: 67–82), which was older than the 1L PBC cohort at 65 years (IQR: 59–74), the 1L PBC + avelumab 1LM cohort at 66 years (IQR: 60–73), and the 1L other therapies cohort at 69 years (IQR: 62–77).

Most patients (56.2%, 1545 of 2820) resided in urban areas, defined as large, medium, or small metropolitan counties per NCHS. The proportion of patients with commercial, Medicare Advantage, and Medicare supplemental health plans, respectively, was 65.1%, 25.9%, and 9.0% for 1L PBC; 65.0%, 26.8%, 8.3% for 1L PBC + avelumab 1LM; 39.6%, 21.2%, 39.2% for 1L IO; and 53.9%, 28.4%, and 17.7% for 1L other therapies. Overall, the mean area-level unemployment rate was 5%, the poverty rate was 10%, and education attainment of high school and above was over 91%. The median area-level family income ranged between USD 84,803 (IQR: USD 61,875–USD 115,245) in the 1L PBC cohort and USD 94,154 (IQR: USD 70,552–USD 123,548) in the 1L IO cohort. Most of the overall cohort had a higher SES than the average of the US population, and 13.7% were in the bottom 25% of the SES index (Table 1).

### 3.3. Baseline Clinical Characteristics

Patient clinical characteristics are reported in Table 2. Of the patients with Eastern Cooperative Oncology Group performance score (ECOG PS) records, the proportion with ECOG PS of 0–1 was 93.5% (578 of 618) in the 1L PBC cohort, 94.9% (93 of 98) in the 1L PBC + avelumab 1LM cohort, 86.8% (367 of 423) in the 1L IO cohort, and 92.6% (301 of 325) in the 1L other therapies cohort.

The mean modified Quan–Charlson Comorbidity Index (QCI) score was higher in the 1L IO cohort than in the other cohorts: 1.75 (SD 1.63) in IL IO, 1.37 (SD 1.49) in 1L PBC, 1.40 (SD 1.56) in 1L PBC + avelumab 1LM, and 1.51 (SD 1.56) in 1L other therapies. Furthermore, compared with the other treatment groups, the 1L IO cohort had higher proportions of patients with chronic pulmonary disease (29.9% vs. 25.5–29.3%), diabetes without chronic complications (26.2% vs. 17.8–25.4%), renal disease (41.0% vs. 23.0–26.8%), peripheral vascular disease (26.8% vs. 18.5–21.0%), and diabetes with chronic complications (15.9% vs. 7.6–12.1%).

### 3.4. Treatment Patterns

In total, 32.8% of patients (926 of 2820) who initiated 1L systemic treatment received 2L treatment, and 10.6% (299 of 2820) received third-line (3L) treatment.

**Figure 2:**
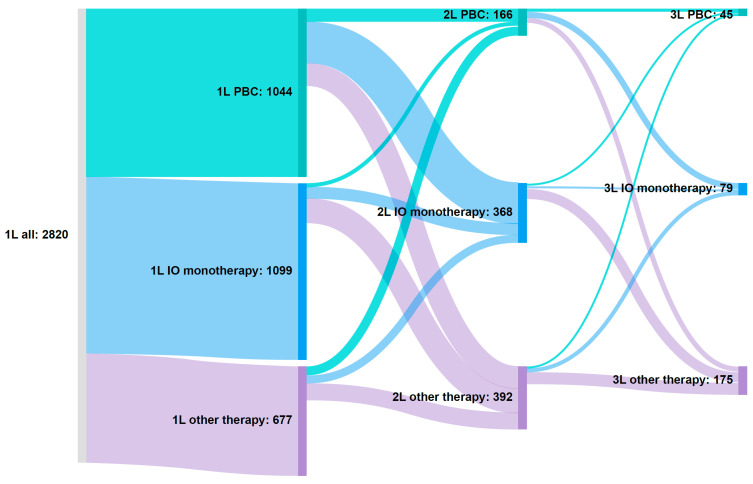
Sankey diagram illustrating treatment patterns. 1L, first line; 2L, second line; 3L, third line; EV, enfortumab vedotin; IO, immuno-oncology; PBC, platinum-based chemotherapy. Patients on therapy at each line of therapy: 1L, n = 2820; 2L, n = 926; 3L, n = 299. 1L other therapies breakdown: gemcitabine (36.3%, 246 of 677), docetaxel (17.1%, 116 of 677), EV monotherapy (6.5%, 44 of 677), docetaxel + gemcitabine (6.5%, 44 of 677), carboplatin + atezolizumab (5.8%, 39 of 677), doxorubicin (5.8%,39 of 677), paclitaxel (4.9%, 33 of 677), carboplatin + paclitaxel + pembrolizumab (4.0%, 27 of 677), carboplatin + pembrolizumab (3.2%, 22 of 677), EV + pembrolizumab (2.7%, 18 of 677), gemcitabine + paclitaxel (2.1%, 14 of 677), and paclitaxel + pembrolizumab (1.2%, 8 of 677). Other additional regimens have <5 patients, and according to Carelon Research policy, any cell with a value of <5 cannot be reported, to limit the re-identification of patients.

Transitions to other treatment options over time, up to a third LOT, are illustrated using a Sankey diagram (Figure 2). Among patients receiving 1L PBC, 45.8% (478 of 1044) received 2L treatment, of whom 85.4% (408 of 478) received 1L PBC only + any 2L option and 14.6% (70 of 478) received 1L PBC and avelumab 1LM + any 2L option (Figure 2). Of patients originally receiving 1L PBC, 24.1% (252 of 1044) initiated 2L IO monotherapy, 13.3% (139 of 1044) initiated other therapies, and 8.3% (87 of 1044) stayed on PBC in 2L (Figure 2).

Of those receiving 1L IO monotherapy, 22.2% of patients (244 of 1099) received any 2L treatment option (Table 3, Figure 2). Specifically, 6.4% of patients (71 of 1099) either stayed on the same IO monotherapy or switched to a different IO monotherapy, 13.9% (153 of 1099) initiated other 2L therapies, and 1.8% (20 of 1099) initiated 2L PBC (Figure 2).

Among patients in the 1L other therapies cohort, 30.1% (204 of 677) received any 2L therapy (Table 3, Figure 2). Specifically, 6.6% (45 of 677) initiated 2L IO monotherapy, 14.8% (100 of 677) initiated other 2L therapies, and 8.7% (59 of 677) initiated 2L PBC (Figure 2).

The most common choices for 3L treatment included EV, sacituzumab govitecan, and erdafitinib (Figure 2).

### 3.5. Clinical Outcomes

The median follow-up time in months for cohorts was 11.2 (IQR: 5.6–20.3) for 1L PBC, 14.6 (IQR: 9.2–21.3) for 1L PBC + avelumab 1LM, 8.6 (IQR: 4.0–17.7) for 1L IO, and 10.1 (IQR: 4.6–21.5) for 1L other therapies. The median treatment-free interval between the end of 1L PBC and start of avelumab 1LM was 2.7 weeks (IQR: 1.1–4.6). Median TTNT in months was 4.8 (IQR: 2.8–8.3), 7.6 (IQR: 6.2–12.3), 5.5 (IQR: 2.8–11.0), and 4.1 (IQR: 1.4–7.6) for 1L PBC, 1L PBC + avelumab 1LM, 1L IO, and 1L other therapies, respectively.

**Figure 3:**
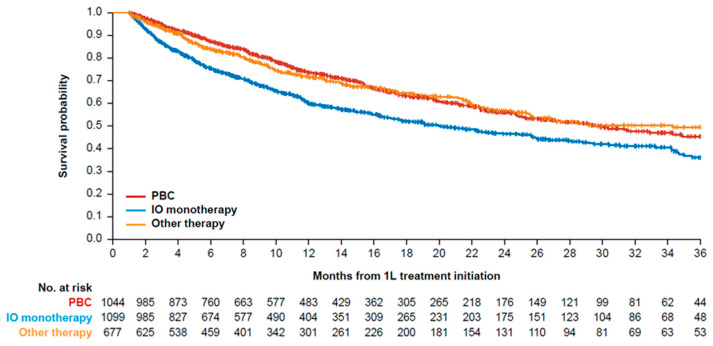
Kaplan–Meier curve of OS for 1L treatment cohorts. 1L, first line; IO, immuno-oncology; OS, overall survival; PBC, platinum-based chemotherapy.

**Figure 4:**
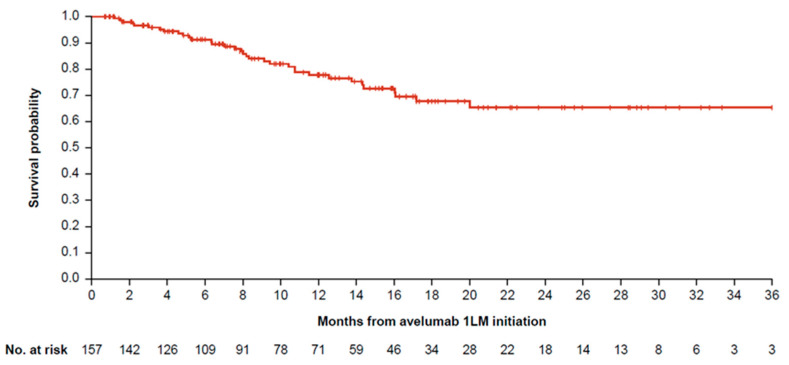
OS for patients with la/mUC who received 1L PBC and avelumab 1LM. 1L, first line; 1LM, first-line maintenance; la/mUC, locally advanced or metastatic urothelial carcinoma; OS, overall survival; PBC, platinum-based chemotherapy.

Median OS in months was 29.7 (95% confidence interval [CI]: 25.1–37.2), 20.0 (95% CI: 17.1–25.6), and 34.3 (95% CI: 25.6–not estimable) for 1L PBC, 1L IO, and 1L other therapies, respectively (Table 3, Figure 3). Median OS for 1L PBC + avelumab 1LM was not estimable because >50% of patients remained alive at the end of the study period (Table 3, Figure 4). Median time on avelumab 1LM treatment was 5.0 months (IQR: 1.8–10.2). OS rates at 1 and 2 years from the start of avelumab 1LM were 78.0% and 65.0%, respectively, while OS rates at 1 and 2 years from the start of 1L PBC were 84.0% and 68.0%, respectively (Table 3, Figure 4).

**Table 3:** Survival rates for patients with la/mUC who received 1L systemic anticancer treatments.

	1L PBC * (n = 1044)	1L IO Monotherapy (n = 1099)	1L Other Therapies (n = 677)	1L PBC + Avelumab 1LM ^#^; OS From 1L PBC Initiation (n = 157)	1L PBC + Avelumab 1LM ^#^; OS From Avelumab Initiation (n = 157)
**OS rates (95% CI)**					
1 year	0.74 (0.71–0.77)	0.60 (0.57–0.63)	0.72 (0.68–0.76)	0.84 (0.78–0.91)	0.78 (0.70–0.86)
2 years	0.56 (0.52–0.60)	0.47 (0.43–0.51)	0.57 (0.52–0.62)	0.68 (0.58–0.78)	0.65 (0.56–0.77)
3 years	0.45 (0.40–0.51)	0.36 (0.32–0.41)	0.49 (0.44–0.55)	0.65 (0.55–0.77)	0.65 (0.56–0.77)
**Median OS, months (95% CI)**	29.7 (25.1–37.2)	20.0 (17.1–25.6)	34.3 (25.6–NE)	NE (NE–NE)	NE (NE–NE)

1L, first line; 1LM, first-line maintenance; CI, confidence interval; IO, immuno-oncology; la/mUC, locally advanced or metastatic urothelial carcinoma; NE, not estimable; OS, overall survival; PBC, platinum-based chemotherapy. * 1L PBC cohort refers to all patients on carboplatin, cisplatin, and dose-dense methotrexate, vinblastine, doxorubicin, and cisplatin systemic therapies. ^#^ 1L PBC + avelumab 1LM is a subgroup of 1L PBC overall.

### 3.6. All-Cause and UC-Related HCRU and Costs

**Figure 5:**
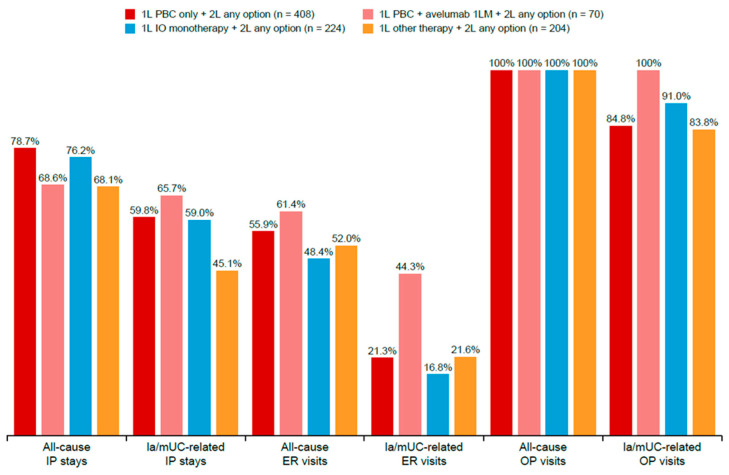
Proportion of patients who received 2L treatment with HCRU. 1L, first line; 1LM, first-line maintenance; 2L, second line; ER, emergency room; HCRU, healthcare resource utilization; IO, immuno-oncology; IP, inpatient; UC, urothelial carcinoma; OP, outpatient; PBC, platinum-based chemotherapy.

The HCRU and costs analysis focused on the 926 patients who received subsequent 2L treatment. Among patients treated with 1L PBC, 1L PBC + avelumab 1LM, 1L IO monotherapy, and 1L other therapies, and who received 2L treatment, the proportion with all-cause inpatient stays were 78.7%, 68.6%, 76.2%, and 68.1%, respectively (Figure 5). The mean all-cause inpatient stays PPPM were lowest in the 1L PBC + avelumab 1LM cohort at 0.13 (SD 0.16), compared with 0.20 (SD 0.26) in 1L PBC, 0.18 (SD 0.25) in 1L IO monotherapy, and 0.18 (SD 0.23) in 1L other therapies cohorts (Table 4).

The proportion of patients with all-cause ER visits was 55.9%, 61.4%, 48.4%, and 52.0% for 1L PBC, 1L PBC + avelumab 1LM, 1L IO monotherapy, and 1L other therapies cohorts, respectively (Figure 5). The mean number of all-cause ER visits PPPM was 0.09 (SD 0.15), 0.11 (SD 0.17), 0.06 (SD 0.10), and 0.08 (SD 0.14) for 1L PBC, 1L PBC + avelumab 1LM, 1L IO monotherapy, and 1L other therapies, respectively (Table 4). The mean number of all-cause outpatient visits was 10.9 (SD 5.7), 10.9 (SD 4.1), 10.0 (SD 4.7), and 10.0 (SD 6.8) for 1L PBC, 1L PBC + avelumab 1LM, 1L IO monotherapy, and 1L other therapies, respectively (Table 4). All patients (100%) had ≥1 all-cause outpatient visit; the proportion of patients with UC-related outpatient visits was 84.8%, 100%, 91.0%, and 83.8% for 1L PBC, 1L PBC + avelumab 1LM, 1L IO, and 1L other therapies cohorts, respectively (Figure 5).

Among patients receiving 2L treatment, median all-cause total direct medical costs PPPM (in 2023 US dollars) for 1L PBC, 1L PBC + avelumab 1LM, 1L IO monotherapy, and 1L other therapies were USD 15,859 (IQR: USD 8400–USD 27,121), USD 19,781 (IQR: USD 14,134–USD 30,550), USD 11,346 (IQR: USD 3164–USD 23,402), and USD 9516 (IQR: USD 2422–USD 21,625), respectively (Table 5). Most costs were attributed to all-cause outpatient visits, with median values of USD 11,464 (IQR: USD 5277–USD 19,057), USD 17,692 (IQR: USD 12,423–USD 25,237), USD 8124 (IQR: USD 2946–USD 18,520), and USD 6347 (IQR: USD 1854–USD 15,740) for 1L PBC, 1L PBC + avelumab 1LM, 1L IO monotherapy, and 1L other therapies, respectively (Table 5). The median costs for all-cause inpatient stays were USD 2320 (IQR: USD 179–USD 6503), USD 674 (IQR: USD 0–USD 3507), USD 464 (IQR: USD 35–USD 3187), and USD 593 (IQR: USD 0–USD 4311) for 1L PBC, 1L PBC + avelumab 1LM, 1L IO monotherapy, and 1L other therapies, respectively (Table 5). The lowest costs in patients who received 2L treatment were those associated with ER visits (Table 5).

## 4. Discussion

This real-world, retrospective cohort study analyzed clinical and economic outcomes of 1L systemic therapies among patients with la/mUC between 2020 and 2023 in the US, using data from the Carelon Research HIRD^®^. The analysis in this study provides insight into the impact of treatment choice on survival outcomes, HCRU, and healthcare costs among patients during this time.

The patients included in this study had a median age of 70 years at bladder cancer diagnosis, which is slightly younger than the US national median age of 73 years [3]; the median age was higher in the 1L IO and other therapies cohorts compared with the 1L PBC and 1L PBC + avelumab 1LM cohorts. Demographically, patients in the 1L PBC + avelumab 1LM cohort were similar in terms of age, insurance type, and geographic region of residence compared with the overall 1L PBC cohort; however, they were slightly younger (median age: 66 years) than those in avelumab 1LM cohorts of previous studies [29,30].

SDoH are known to influence outcomes and treatment in patients with bladder cancer [31]. In this analysis, two-thirds of the 1L PBC + avelumab 1LM cohort were distributed in the top 50% of the SES index categories, while about 57% of the overall 1L PBC cohort were in higher SES categories. Differences between cohorts were seen by race, with a higher percentage of White patients, and fewer Black patients, in the 1L PBC + avelumab 1LM cohort than in the overall 1L PBC cohort, similar to that seen in Bakaloudi et al. (92.6% White, 5.6% non-White) [30]. Hasan et al. also reported that female sex and Black race were associated with a lower likelihood of receiving National Comprehensive Cancer Network (NCCN)-recommended treatment than male sex and White race [31]. Sex-related differences were observed within the current population, with female patients being more likely to receive 1L PBC than male patients.

Clinical characteristics showed comparable QCI and ECOG PS across the study cohorts. Among patients with available ECOG PS, most (91%) had an ECOG PS of 0–1, though a lower proportion of patients in the 1L IO cohort had a score of 0–1 than in the 1L PBC cohort (33.4% vs. 55.4%). Considering the potential predictive impact of ECOG PS ≥2 on survival outcomes, ECOG PS plays a role in treatment choice [1]. In the present IMPACT UC-III study, QCI scores were calculated excluding solid tumors and hematological malignancies. The overall mean QCI score of 1.6 was similar to that of 1.1 reported by Grivas et al. [32]. In the present study, QCI scores were higher in the 1L IO and 1L other therapies cohorts than in the 1L PBC and 1L PBC + avelumab 1LM groups but were similar in the 1L PBC + avelumab 1LM and 1L PBC cohorts.

The presence of comorbidities also plays a significant role in the choice of 1L treatment. Compared with the 1L PBC cohort, higher proportions of patients in the 1L IO and 1L other therapies cohorts were impacted by comorbidities. The oldest patients with the most comorbidities were concentrated in the 1L IO cohort, while among patients receiving 1L PBC, the youngest and healthiest patients were concentrated in the DDMVAC cohort. This is consistent with analyses from a similar real-world population [33]. Compared with the 1L PBC cohort, the 1L IO cohort had higher proportions of patients with chronic pulmonary disease, diabetes with and without chronic complications, and peripheral vascular disease. Furthermore, the proportion of patients with renal disease was highest in the 1L IO cohort, with significant differences observed between the 1L IO (41.0%) and 1L PBC (23.0%) cohorts. These variations in clinical characteristics suggest that the presence of comorbidities and renal impairment influenced the choice of 1L systemic therapy. This is supported by the fact that cisplatin is contraindicated for patients with renal impairment, as previously defined using the Galsky criteria to assess cisplatin ineligibility for bladder cancer [6].

The proportion of patients initiated on 1L PBC therapies in this study (37.0%) was consistent with other published literature in the real-world setting [33,34,35,36]. The current IMPACT UC-III study results demonstrated a higher proportion of 1L IO monotherapy use (39.0%) compared with a study in patients with la/mUC by Geynisman et al. (24.1%; N = 8183) [34], the IMPACT UC-I real-world study (20.0%; N = 8630) [35], and the IMPACT UC-II real-world study (27.3%; N = 3006) [36]. The period of this study (January 2020–July 2023) was later than these prior real-world studies and included patients from the period after the approval of avelumab 1LM by the FDA; therefore, it captured the availability and wider adoption of IO in 2020 and beyond.

Treatment duration in the current study was consistent with that seen in the published literature. In the IMPACT UC-II real-world study, Bilen et al. found a median treatment duration of 4.3 months in carboplatin-based 1L recipients and 4.0 months in those receiving cisplatin-based 1L treatment [36]. In a population of patients who did not respond to 1L chemotherapy in the US Oncology Network, the median time to treatment failure was 2.5 months in the group receiving 1L chemotherapy to 2L IO, and 2.3 months in patients receiving 1L to 2L chemotherapy [28]. In patients with unresectable la/mUC in Canada, the median time on treatment for 1L systemic therapy was 2.8 months [37].

Median OS for the 1L treatment groups varied by treatment type and was higher than in previous real-world studies of patients with la/mUC [33,35,38]. Patients treated with 1L PBC had longer median OS than those on 1L IO monotherapy, a trend also seen in patients starting avelumab 1LM after completing 1L PBC. The results from the current IMPACT UC-III study corroborates IMPACT UC-I study results, which reported higher median OS in patients receiving 1L PBC than those on 1L IO monotherapy [35]. Similarly, Kirker et al. reported longer median OS for 1L PBC (18.3 months) compared to IL IO (14.6 months) therapies from the Flatiron Health database (2015–2021) [38]. Additionally, Gupta et al. reported longer median OS in 1L PBC compared to PBC-ineligible groups (13.3 vs. 5.1 months) [33]. Other real-world studies reported median OS ranging from 10.6 to 26.2 months from avelumab 1LM initiation [13,39,40,41,42,43].

Using US-based data from Flatiron, Tempus, and PATRIOT II studies, the median OS from avelumab 1LM initiation was reported as 23.8, 20.6, and 24.4 months, respectively [39,40,41]. In the JAVELIN Bladder 100 trial, with long-term follow-up (data cutoff: June 2021), median OS with avelumab was 23.8 months (95% CI: 19.9–28.8) versus 15.0 with control (HR: 0.76), and median investigator-assessed PFS was 5.5 versus 2.1 months, respectively (HR: 0.54) [13]. Comparable median OS results were reported in other real-world studies conducted in Italy (READY CUP; 26.2 months) and France (AVENANCE; 21.1 months) [42,44], further supporting the real-world effectiveness of avelumab 1LM and the JAVELIN Bladder 100 trial results [13]. The observed higher median OS across all treatment cohorts in the current study may have been influenced by several factors, including the younger median age, the population’s commercially insured status, and the availability of newer subsequent therapies.

Only one-third of patients in the IMPACT UC-III study who received 1L systemic treatment went on to receive 2L treatment. These results align with previous findings that most patients with la/mUC in real-world settings do not initiate later LOTs [8,45]. This finding highlights the importance of appropriate treatment optimization at 1L and the need to use the most effective agents at an early stage. Although treatment rates in 2L remained low in this study, we evaluated HCRU and direct healthcare costs across the 1L therapy cohorts for patients who received 2L treatment, as treatment and costs associated with HCRU are known to increase as the disease progresses [5].

All patients had one or more all-cause outpatient visits. The number of outpatient visits did not vary greatly among groups; however, ER visits, inpatient stays, and medical costs were impacted by treatment group. The treatment of la/mUC was associated with substantial HCRU and costs, driven primarily by outpatient visits. Compared with patients who received 1L treatment only, patients who received 2L treatment had higher rates of all-cause and UC-related inpatient hospitalization, ER visits, and outpatient visits. UC-related utilization contributed to all-cause medical costs, with outpatient visits being the primary cost driver, consistent with other studies [36,46].

Avelumab 1LM slightly increased costs in the 1L PBC + avelumab 1LM cohort compared with those in the 1L PBC cohort. This was mainly driven by outpatient and treatment costs; however, mean inpatient stays and length of stay were lower among patients treated in the 1L PBC + avelumab 1LM cohort compared with patients in the 1L IO monotherapy cohort. In the PATRIOT II study, both hospitalizations and the incidence of ER visits were higher during, and 90 days after the avelumab treatment period than during the 1L PBC treatment period [40]. In a cost analysis study among patients with la/mUC, costs per patient were lower with 1L PBC + avelumab 1LM than with EV + pembrolizumab and nivolumab + cisplatin/gemcitabine [47]. A modeling exercise of optimal treatment sequences also found a survival benefit with 2L EV following PBC + avelumab 1LM and that this sequence was associated with substantially lower costs versus 1L EV + pembrolizumab in platinum-eligible patients with la/mUC [48].

In summary, treatment choice can be influenced by baseline patient clinical characteristics and prognostic factors (i.e., ECOG PS, medical comorbidities such as cardiovascular diseases and renal dysfunction, SDoH, prior perioperative systemic treatment, and visceral metastases). Physician, patient, and caregiver preferences were not evaluated in the current IMPACT UC-III study, but these could influence treatment choices. In addition, the selected health insurance plan could influence treatment choice. In the current study, patients with commercial insurance health plans were more likely to receive 1L PBC than patients with Medicare health plans, which aligns with an analysis by Hasan et al., who reported that patients with UC who had private insurance were more likely to receive NCCN-recommended treatments [31]. The results from IMPACT UC-III provide a baseline assessment of clinical and economic data in the real world among patients with la/mUC as the treatment landscape continues to evolve. Further real-world studies are needed to assess the clinical and economic impacts of newer and recently approved treatments, as well as contemporary treatment sequences.

### Study Limitations

As with all studies, there are limitations to this analysis. The population data assessed in this analysis were collected from commercially insured patients in the US, Medicare Advantage enrollees, patients with supplemental Medicare health plan, and those with available clinical data from HIRD^®^. This may limit the generalizability of these results to other population segments, such as those receiving traditional fee-for-service Medicare, the uninsured, and untreated patients. Furthermore, a larger proportion of patients in the HIRD^®^ are commercially insured and are, therefore, likely to be younger than the general population. CCQP offers incentives to physicians for treating according to evidence-based guidelines created by the health plans participating in the program, which could have influenced treatment choices for these patients. Administrative claims data are primarily collected for billing and reimbursement purposes and are subject to potential coding biases, inconsistencies, and missing data. In addition, some costs, (e.g., expenses incurred by patients with Medicare receiving hospice care) may have been missed in our analysis if these were paid in full by the Centers for Medicare and Medicaid Services.

An algorithm was used to define LOTs. In addition, we could not identify response data or patient eligibility for 1LM. Avelumab use in 2L could not be differentiated from 1LM using the LOT algorithm. Because disease staging was available only for patients with CCQP data, we used International Classifications of Diseases 10th Revisions secondary malignant neoplasm codes to define patients with la/mUC.

Clinical trial participants often receive treatments or combinations of therapies not commonly available in regular practice. Therefore, they were excluded to preserve the integrity, applicability, and generalizability of the real-world research findings. While this exclusion may introduce selection or survivorship bias, the study’s descriptive nature focused on understanding real-world treatment patterns, making sensitivity and comparative analyses unnecessary. No evidence is available on whether any patients went on to participate in a clinical trial.

Although mortality was identified in the HIRD^®^ through a combination of sources including inpatient discharge status from claims, reason for insurance disenrollment, linking to obituary data, and linking to the DMF, all deaths may not have been captured [49]. Nonetheless, a mortality validation study reported that the HIRD’s composite death measure has a sensitivity of 88.9% (95% CI = 88.5%, 89.3%), specificity of 89.1% (95% CI = 88.6%, 89.6%), a positive predictive value of 93.4% (95% CI = 93.1%, 93.7%), and a negative predictive value of 82.3% (95% CI = 81.7%, 82.9%) when compared to the National Death Index.

The ECOG score serves as an approximate measure of health status, but it was missing or unknown for 52% of patients. While unobserved health status or other patient characteristics that correlate with treatment selection and outcomes may introduce confounding variables, it is important to note that this was a descriptive study. Its primary objective was to understand the real-world treatment patterns of patients with bladder cancer in the United States.

The median follow-up time in months for cohorts was 11.2 for 1L PBC, 14.6 for 1L PBC + avelumab 1LM, 8.6 for 1L IO, and 10.1 for 1L other therapies. Indeed, the modest follow-up duration limits our ability to interpret long-term survival outcomes. Future studies with extended follow-up periods would provide more comprehensive insights into survival outcomes.

This analysis presents a descriptive summary of the final study cohort and subgroups only, with no comparative analysis and no adjustment for baseline characteristics. Assessments are limited to treatments approved in the US and incorporated into clinical guidelines during the study period and may not reflect more recent changes in the NCCN guidelines. The study time period only extended until July 2023, therefore limiting the ability to assess the impact of recently approved novel treatments. This study included direct medical costs only, so patient out-of-pocket and indirect costs are not included in the analysis.

## 5. Conclusions

The IMPACT UC-III study examined patient demographics, clinical characteristics, treatment patterns, OS, TTNT, HCRU, and cost outcomes among patients with la/mUC in the real-world setting who received 1L systemic therapy from January 2020 to July 2023 in the US. High attrition was seen across LOTs, demonstrating the importance of appropriate 1L treatment selection for care optimization. Survival rates were higher than those in the published literature and varied based on the type of 1L treatment received. Patients treated with 1L PBC, including those who received avelumab 1LM, had longer OS than those treated with 1L IO monotherapy. HCRU and direct medical costs were substantial among patients with la/mUC who received 2L treatment, including high rates of hospital admissions and outpatient visits, resulting in considerable total costs.

The choice of treatment for la/mUC is influenced by many factors, but patient clinical characteristics and comorbidities are key. The treatment landscape of la/mUC continues to evolve, with new therapies being introduced and the rapid expansion of available 1L and 2L treatment options. The results from this study highlight the need for additional studies with more recent data and longer follow-up to assess the clinical and economic impact of novel regimens and treatment sequences in la/mUC and to optimize care protocols for managing this aggressive form of cancer.

## Figures and Tables

**Table 1:** Baseline demographic and SDoH characteristics.

Characteristic	Overall, All Cohorts (N = 2820)	1L PBC * (n = 1044)	1L PBC + Avelumab 1LM ^#^ (n = 157)	1L IO Monotherapy (n = 1099)	1L Other Therapies (n = 677)
**Age at index, median (IQR), years**	70 (62–79)	65 (59–74)	66 (60–73)	76 (67–82)	69 (62–77)
**Male sex, n (%)**	1951 (69.2)	664 (63.6)	125 (79.6)	791 (72.0)	496 (73.3)
**Payor type, n (%)**					
Commercial health plan	1480 (52.5)	680 (65.1)	102 (65.0)	435 (39.6)	365 (53.9)
Medicare Advantage	695 (24.7)	270 (25.9)	42 (26.8)	233 (21.2)	192 (28.4)
Medicare Other (supplemental)	645 (22.9)	94 (9.0)	13 (8.3)	431 (39.2)	120 (17.7)
**Race/ethnicity, n (%)**					
Asian, not Hispanic or Latino	59 (2.1)	23 (2.2)	<5 **^‡^**	23 (2.1)	13 (1.9)
Black or African American, not Hispanic or Latino	112 (4.0)	52 (5.0)	<5 **^‡^**	31 (2.8)	29 (4.3)
Hispanic or Latino of any race	108 (3.8)	48 (4.6)	8 (5.1)	30 (2.7)	30 (4.4)
White, not Hispanic or Latino	2058 (73.0)	767 (73.5)	123 (78.3)	783 (71.2)	508 (75.0)
Other race, not Hispanic or Latino, including American Indian or Alaska Native and Native Hawaiian or other Pacific Islander	23 (0.8)	9 (0.9)	<5 **^‡^**	8 (0.8)	6 (0.9)
Unknown or undisclosed	460 (16.3)	145 (13.9)	19 (12.1)	224 (20.4)	91 (13.4)
**Area-level SES index category, n (%)**					
1 (bottom 25% of SES index score)	372 (13.2)	165 (15.8)	16 (10.2)	108 (9.8)	99 (14.6)
2	660 (23.4)	268 (25.7)	34 (21.7)	249 (22.7)	143 (21.1)
3	796 (28.2)	305 (29.2)	55 (35.0)	309 (28.1)	182 (26.9)
4 (top 25% of SES index score)	895 (31.7)	262 (25.1)	45 (28.7)	407 (37.0)	226 (33.4)
Missing/unknown	97 (3.4)	44 (4.2)	7 (4.5)	26 (2.4)	27 (4.0)
**Area-level SES index components**					
Unemployment rate, mean (SD) ^a^	0.05 (0.05)	0.05 (0.05)	0.04 (0.04)	0.05 (0.05)	0.05 (0.06)
Poverty rate, mean (SD) ^b^	0.10 (0.10)	0.11 (0.11)	0.09 (0.08)	0.09 (0.10)	0.10 (0.10)
Family income, median (IQR) ^c^	USD 89,108 (USD 66,250–USD 120,375)	USD 84,803 (USD 61,875–USD 115,245)	USD 89,293 (USD 71,023–USD 115,262)	USD 94,154 (USD 70,552–USD 123,548)	USD 88,750 (USD 65,326–USD 122,292)
Home value, median (IQR) ^d^	USD 237,150 (USD 149,175–USD 402,150)	USD 216,900 (USD 138,600–USD 356,700)	USD 235,100 (USD 157,700–USD 370,150)	USD 252,900 (USD 163,425–USD 452,100)	USD 246,800 (USD 149,100–USD 430,900)
Rate of no high school diploma, mean (SD) ^e^	0.07 (0.08)	0.08 (0.08)	0.08 (0.08)	0.07 (0.07)	0.07 (0.09)
Rate of college degree, mean (SD) ^f^	0.35 (0.21)	0.32 (0.20)	0.34 (0.19)	0.37 (0.21)	0.36 (0.22)
Crowding ^g^	0.02 (0.05)	0.02 (0.05)	0.02 (0.04)	0.02 (0.04)	0.02 (0.05)
**Individual-level residency urbanicity level, n (%)**					
Urban	1545 (54.8)	546 (52.3)	72 (45.9)	633 (57.6)	366 (54.1)
Suburban	711 (25.2)	259 (24.8)	45 (28.7)	269 (24.5)	183 (27.0)
Rural	491 (17.4)	205 (19.6)	34 (21.7)	178 (16.2)	108 (16.0)
Missing/unknown	73 (2.6)	34 (3.3)	6 (3.8)	19 (1.7)	20 (3.0)
**Area-level English spoken less than “well,”mean (SD)** ^h^	0.02 (0.05)	0.02 (0.05)	0.02 (0.04)	0.02 (0.05)	0.03 (0.05)
**Area-level education attainment high school or above, mean (SD)** ^i^	0.91 (0.09)	0.90 (0.09)	0.91 (0.08)	0.92 (0.08)	0.91 (0.09)

1L, first line; 1LM, first-line maintenance; IO, immuno-oncology; IQR, interquartile range; PBC, platinum-based chemotherapy; SD, standard deviation; SDoH, social determinants of health; SES, socioeconomic status. * 1L PBC cohort refers to all patients on carboplatin, cisplatin, and dose-dense methotrexate, vinblastine, doxorubicin, and cisplatin systemic therapies. ^#^ 1L PBC + avelumab 1LM is a subgroup of 1L PBC overall. **^‡^** According to Carelon Research policy, any cell with a value of 1–5 or any cell that allows a value of 1–5 to be derived from other reported cells or information cannot be reported. ^a^ Proportion of civilian labor force population aged ≥16 years who are unemployed. ^b^ Proportion of population with incomes < 100% of the federal poverty level. ^c^ Median family income (in 2020 inflation-adjusted dollars). ^d^ Median home value among owner-occupied housing units. ^e^ Proportion of population aged ≥25 years who have less than a 12th-grade education. ^f^ Proportion of population aged ≥25 years who have at least 4 years of college. ^g^ Proportion of households with more than one person per room (including both owner-occupied and renter-occupied housing units). ^h^ Proportion of population (persons aged ≥5 years) who speak English less than “well.” ^i^ Proportion of population aged ≥25 years who have a high school degree/general education diploma or above.

**Table 2:** Baseline clinical characteristics.

Characteristic	Overall, All Cohorts (N = 2820)	1L PBC * (n = 1044)	1L PBC + Avelumab 1LM ^#^ (n = 157)	1L IO Monotherapy (n = 1099)	1L Other Therapies (n = 677)
**ECOG performance score, n (%)**					
0	562 (19.9)	255 (24.4)	41 (26.1)	154 (14.0)	153 (22.6)
1	684 (24.3)	323 (30.9)	52 (33.1)	213 (19.4)	148 (21.9)
≥2	120 (4.3)	40 (3.8)	5 (3.2)	56 (5.1)	24 (3.5)
Missing/unknown	1454 (51.6)	426 (40.8)	59 (37.6)	676 (61.5)	352 (52.0)
**QCI, mean (SD)**	1.6 (1.57)	1.37 (1.49)	1.4 (1.6)	1.75 (1.63)	1.51 (1.56)
**Comorbidities, n (%)**					
Congestive heart failure	348 (12.3)	103 (9.9)	13 (8.3)	170 (15.5)	75 (11.1)
Peripheral vascular disease	644 (22.8)	207 (19.8)	29 (18.5)	295 (26.8)	142 (21.0)
Cerebrovascular disease	321 (11.4)	80 (7.7)	11 (7.0)	165 (15.0)	76 (11.2)
Chronic pulmonary disease	815 (28.9)	289 (27.7)	40 (25.5)	328 (29.9)	198 (29.3)
Diabetes without chronic complications	715 (25.4)	255 (24.4)	28 (17.8)	288 (26.2)	172 (25.4)
Diabetes with chronic complications	375 (13.3)	118 (11.3)	12 (7.6)	175 (15.9)	82 (12.1)
Renal disease	853 (30.3)	240 (23.0)	42 (26.8)	451 (41.0)	162 (23.9)
Mild liver disease	635 (22.5)	235 (22.5)	37 (23.6)	226 (20.6)	174 (25.7)
Patients with surgical procedures	1291 (45.8)	559 (53.5)	92 (58.6)	448 (40.8)	284 (41.9)

1L, first line; 1LM, first-line maintenance; ECOG, Eastern Cooperative Oncology Group; IO, immuno-oncology; PBC, platinum-based chemotherapy; QCI, Quan–Charlson Comorbidity Index; SD, standard deviation. * 1L PBC cohort refers to all patients on carboplatin, cisplatin, and dose-dense methotrexate, vinblastine, doxorubicin, and cisplatin systemic therapies. ^#^ 1L PBC + avelumab 1LM is a subgroup of 1L PBC overall.

**Table 4:** HCRU PPPM among patients with UC who received 2L treatment.

Mean (SD)	1L PBC without Avelumab 1LM + 2L Any Option (n = 408)	1L PBC + Avelumab 1LM + 2L Any Option (n = 70)	1L IO Monotherapy + 2L Any Option (n = 244)	1L Other Therapy + 2L Any Option (n = 204)
All-cause IP stays	0.20 (0.26)	0.13 (0.16)	0.18 (0.25)	0.18 (0.23)
UC-related IP stays	0.13 (0.22)	0.12 (0.15)	0.13 (0.21)	0.11 (0.20)
All-cause ER visits	0.09 (0.15)	0.11 (0.17)	0.06 (0.10)	0.08 (0.14)
UC-related ER visits	0.03 (0.10)	0.06 (0.14)	0.02 (0.06)	0.03 (0.09)
All-cause OP visits	10.90 (5.72)	10.87 (4.07)	10.00 (4.67)	9.95 (6.82)
UC-related OP visits	4.62 (4.71)	7.19 (3.02)	4.47 (3.68)	4.07 (5.70)

1L, first line; 1LM, first-line maintenance; 2L, second line; ER, emergency room; HCRU, healthcare resource utilization; IO, immuno-oncology; IP, inpatient; OP, outpatient; PBC, platinum-based chemotherapy; PPPM, per patient per month; SD, standard deviation; UC, urothelial carcinoma.

**Table 5:** Direct healthcare costs* PPPM among patients with UC who received 2L treatment (USD) *.

Median (IQR), USD	1L PBC without Avelumab 1LM + 2L Any Option (n = 408)	1L PBC + Avelumab 1LM + 2L Any Option (n = 70)	1L IO Monotherapy + 2L Any Option (n = 244)	1L Other Therapy + 2L Any Option (n = 204)
All-cause IP stays	2320 (179–6503)	674 (0–3507)	464 (35–3187)	593 (0–4311)
UC-related IP stays	738 (0–4301)	566 (0–2732)	146 (0–1796)	0 (0–1654)
All-cause ER visits	43 (0–292)	80 (0–376)	0 (0–76)	7 (0–176)
UC-related ER visits	0 (0–0)	0 (0–124)	0 (0–0)	0 (0–0)
All-cause OP visits	11,464 (5277–19,057)	17,692 (12,423–25,237)	8124 (2946–18,520)	6347 (1854–15,740)
UC-related OP visits	4273 (109–12,273)	14,347 (8887–19,682)	3577 (906–14,976)	988 (36–7594)
All-cause total medical costs	15,859 (8400–27,121)	19,781 (14,134–30,550)	11,346 (3164–23,402)	9516 (2422–21,625)
UC-related total medical costs	7780 (1004–17,978)	17,216 (10,259–24,506)	4700 (1384–18,267)	1568 (170–11,918)

1L, first line; 1LM, first-line maintenance; 2L, second line; ER, emergency room; HCRU, healthcare resource utilization; IO, immuno-oncology; IP, inpatient; IQR, interquartile range; OP, outpatient; PBC, platinum-based chemotherapy; PPPM, per patient per month; UC, urothelial carcinoma. * Health costs are reported PPPM for patients who received 2L treatment and adjusted to 2023 US dollars using the Consumer Price Index from the US Bureau of Labor Statistics.

## Data Availability

The datasets presented in this article are not readily available for use by outside parties because of contractual obligations to the data sources. Requests for data sharing by license can be submitted to data@carelon.com.

## Supplementary Materials

The following supporting information can be downloaded at https://www.mdpi.com/article/10.3390/curroncol32070384/s1, Figure S1. Study design; Table S1: Baseline demographic and SDoH characteristics; Table S2: Baseline clinical characteristics; Data S1: Data sources; Data S2: Baseline clinical characteristics and clinical outcomes by first-line platinum-based chemotherapy (1L PBC) regimen. References [49,50,51] are cited in the supplementary materials.

## Abbreviations

The following abbreviations are used in this manuscript:

1L	first line
1LM	first-line maintenance
2L	second line
3L	third line
ADC	antibody–drug conjugates
CCQP	Cancer Care Quality Program
CI	confidence interval
DDMVAC	dose-dense methotrexate, vinblastine, doxorubicin, and cisplatin
DMF	Death Master File
ECOG PS	Eastern Cooperative Oncology Group performance score
ER	emergency room
EV	enfortumab vedotin
FDA	Food and Drug Administration
HCRU	healthcare resource utilization
HIRD^®^	Healthcare Integrated Research Database
IO	immuno-oncology
IQR	interquartile range
la/mUC	locally advanced/metastatic urothelial carcinoma
LOT	line of therapy
NCCN	National Comprehensive Cancer Network
NCHS	National Center for Health Statistics
NE	not estimable
OP	outpatient
OS	overall survival
PBC	platinum-based chemotherapy
PD-L1	programmed death-ligand 1
PPPM	per patient per month
QCI	Quan–Charlson Comorbidity Index
SD	standard deviation
SDoH	social determinants of health
SES	socioeconomic status
TTNT	time to next treatment
UC	urothelial carcinoma
US	United States
