# Supplementary material for: Real-World Retrospective Study of Clinical and Economic Outcomes Among Patients with Locally Advanced or Metastatic Urothelial Carcinoma Treated with First-Line Systemic Anti-Cancer Therapies in the United States: Results from the IMPACT UC-III Study
Source: Curr Oncol. 2025 Jul 2;32(7):384. doi: 10.3390/curroncol32070384 (PMC12293318; doi:10.3390/curroncol32070384)
Supplement: Supplementary file 1 [file curroncol-32-00384-s001.zip › curroncol-3597079-supplementary.pdf]

Supplement to:

Real-World Retrospective Study of Clinical and Economic Outcomes Among Patients with Locally Advanced or Metastatic Urothelial Carcinoma Treated with First-Line Systemic Anti-Cancer Therapies in the United States: Results from the IMPACT UC-III Study

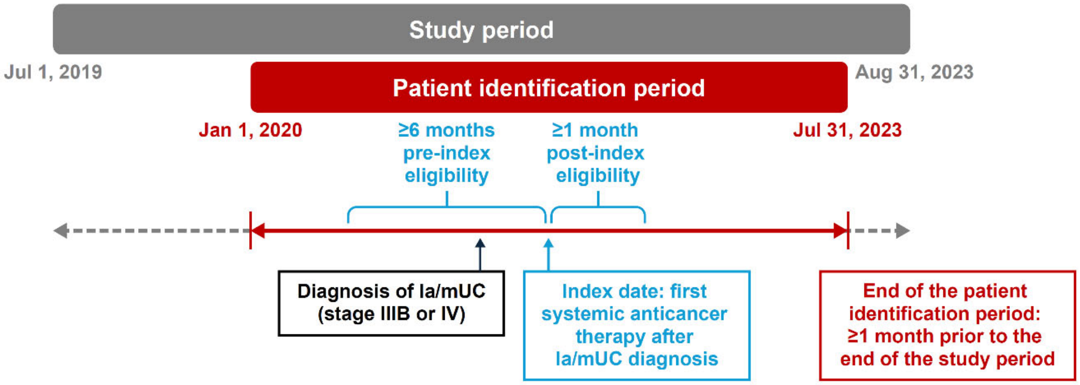

Figure S1. Study design.

Abbreviations: la/mUC, locally advanced/metastatic urothelial carcinoma.

Table S1. Baseline demographic and SDoH characteristics.

	Cisplatin-Based Chemotherapy n = 473	Carboplatin-Based Chemotherapy n = 516	DDMVAC n = 55
**Age at index, median (IQR), years**	63.0 (58.00–70.00)	68.0 (61.00–77.00)	59.0 (54.00–63.00)
**Male sex, n (%)**	333 (70.4)	287 (55.6)	44 (80.0)
**Payor type, n (%)**			
Commercial health plan	340 (71.9)	293 (56.8)	47 (85.5)
Medicare Advantage health plan	105 (22.2)	163 (31.6)	*<5
Medicare Other (supplemental) health plan	28 (5.9)	60 (11.6)	6 (10.9)
**Race/ethnicity, n (%)**			
Asian, not Hispanic or Latino	12 (2.5)	9 (1.7)	*<5
Black or African American, not Hispanic or Latino	23 (4.9)	26 (5.0)	*<5
Hispanic or Latino of any race	27 (5.7)	20 (3.9)	*<5
White, not Hispanic or Latino	338 (71.5)	382 (74.0)	47 (85.5)
Other race, not Hispanic or Latino including American Indian or Alaska Native and Native Hawaiian or other Pacific Islander	6 (1.3)	*<5	0 (0.0)
Unknown or undisclosed	67 (14.2)	76 (14.7)	*<5
**Area-level SES index category, n (%)**			
1 (bottom 25% of SES index score)	79 (16.7)	79 (15.3)	7 (12.7)
2	127 (26.8)	127 (24.6)	14 (25.5)
3	130 (27.5)	160 (31.0)	15 (27.3)
4 (top 25% of SES index score)	117 (24.7)	128 (24.8)	17 (30.9)
Missing/unknown	20 (4.2)	22 (4.3)	*<5
**Area-level SES index components**			
Unemployment rate, mean (SD)[a]	0.05 (0.06)	0.05 (0.05)	0.05 (0.06)
Poverty rate, mean (SD)[b]	0.11 (0.11)	0.11 (0.11)	0.10 (0.13)
Family income, median (IQR)[c]	$83,780 ($60,868–$112,867)	$85,763 ($63,461–$117,053)	$90,871 ($58,750–$115,262)
Home value, median (IQR)[d]	$212,100 ($139,200–$364,950)	$218,350 ($138,300–$345,775)	$256,200 ($144,825–$407,450)
Rate of no high school diploma, mean (SD)[e]	0.09 (0.09)	0.08 (0.08)	0.07 (0.08)
Rate of college degree, mean (SD)[f]	0.31 (0.20)	0.33 (0.20)	0.36 (0.23)
Crowding[g]	0.02 (0.05)	0.02 (0.04)	0.02 (0.04)
**Individual-level residency urbanicity level, n (%)**			
Urban	246 (52.0)	269 (52.1)	31 (56.4)
Suburban	116 (24.5)	133 (25.8)	10 (18.2)
Rural	96 (20.3)	97 (18.8)	12 (21.8)
Missing/unknown	15 (3.2)	17 (3.3)	*<5
**Area-level English spoken less than "well", mean (SD)[h]**	0.02 (0.05)	0.02 (0.04)	0.02 (0.04)

**Area-level education attainment high school or above, mean (SD)**[i]	0.90 (0.10)	0.90 (0.08)	0.92 (0.09)

DDMVAC, dose-dense methotrexate, vinblastine, doxorubicin, and cisplatin; IQR, interquartile range; SD, standard deviation; SDoH, social determinants of health; SES, socioeconomic status.
[a]Proportion of civilian labor force population aged ≥16 years who are unemployed. [b]Proportion of population with incomes <100% of the federal poverty level. [c]Median family income (in 2020 inflation-adjusted dollars). [d]Median home value among owner-occupied housing units. [e]Proportion of population aged ≥25 years who have less than a 12th-grade education. [f]Proportion of population aged ≥25 years who have at least 4 years of college. [g]Proportion of households with more than one person per room (including both owner-occupied and renter-occupied housing units). [h]Proportion of population (persons aged ≥5 years) who speak English less than "well." [i]Proportion of population aged ≥25 years who have a high school degree/general education diploma or above.
*According to Carelon Research policy, any cell with a value of 1–5 or any cell that allows a value of 1–5 to be derived from other reported cells or information cannot be reported.

Table S2. Baseline clinical characteristics

Characteristic, n (%)	Cisplatin-Based Chemotherapy n = 473	Carboplatin-Based Chemotherapy n = 516	DDMVAC n = 55
**ECOG performance score**			
0	125 (26.4)	106 (20.5)	24 (43.6)
1	157 (33.2)	150 (29.1)	16 (29.1)
≥2	12 (4.1)	28 (5.4)	0 (0.0)
Missing/unknown	179 (37.8)	232 (45.0)	15 (27.3)
**Congestive heart failure**	38 (8.0)	61 (11.8)	*<5
**Peripheral vascular disease**	79 (16.7)	119 (23.1)	9 (16.4)
**Cerebrovascular disease**	27 (5.7)	51 (9.9)	*<5
**Chronic pulmonary disease**	126 (26.6)	154 (29.8)	9 (16.4)
**Diabetes without chronic complications**	92 (19.5)	153 (29.7)	10 (18.2)
**Diabetes with chronic complications**	32 (6.8)	83 (16.1)	*<5
**Renal disease**	76 (16.1)	159 (30.8)	5 (9.1)
**Mild liver disease**	114 (24.1)	107 (20.7)	14 (25.5)
**Patients with surgical procedures**	284 (60.0)	201 (39.0)	47 (85.5)

DDMVAC, dose-dense methotrexate, vinblastine, doxorubicin, and cisplatin; ECOG, Eastern Cooperative Oncology Group.
*According to Carelon Research policy, any cell with a value of 1–5 or any cell that allows a value of 1–5 to be derived from other reported cells or information cannot be reported.

Data S1. Data sources.

- Carelon Research Healthcare Integrated Research Database (HIRD®)
    - Patient-level data come from administrative claims integrated across data sources (i.e., professional claims, facility claims, outpatient pharmacy claims, and enrollment information) and years (from 2006 through the most recent calendar quarter) for approximately 90 million members. Data are obtained from health plans across the US and represent members in each of the 50 states.
    - Sociodemographic and social determinants of health (SDoH) data from various sources are integrated into the HIRD. Individual-level race/ethnicity are classified into 8 combined categories, namely:
      1) Hispanic or Latino
      2) Black or African American, not Hispanic or Latino
      3) White, not Hispanic or Latino
      4) Asian, not Hispanic or Latino
      5) Native Hawaiian or other Pacific Islander, not Hispanic or Latino
      6) American Indian or Alaska Native, not Hispanic or Latino
      7) Other race, not Hispanic or Latino and
      8) Unknown or undisclosed race/ethnicity.[1]
    - Area-level data on socioeconomic status (education, income, and employment), housing, transportation, public safety net usage, and race/ethnicity are derived from publicly available data in the American Community Survey (ACS) via 9-digit zip code linkage. The US Census Bureau administers the ACS annually, collecting information from approximately 2.0–2.5 million randomly sampled households per year, and its data are publicly available. ACS indicators in the HIRD represent 5-year averages at the census block group level; specifically, the 2020 ACS data are averages of the 2016–2020 annual estimates. More information can be found here: https://www.census.gov/programs-surveys/acs/data.html
    - Oncology data from the Cancer Care Quality Program (CCQP) are integrated with medical claims data in the HIRD. Detailed clinical oncology data (i.e., cancer stage, pathology/histology, line of treatment, weight and height, and the Eastern Cooperative Oncology Group performance score for individuals who are undergoing cancer treatment in outpatient settings are captured when a healthcare provider submits a request for preauthorization of a cancer treatment. CCQP data have been validated against medical records.[2]
    - Mortality is identified in the HIRD through a combination of inpatient discharge status, reason for disenrollment, third-party obituary data, utilization management data, Center for Medicare and Medicaid Services records, and linkage to the Death Master File (DMF) sourced from the US Social Security Administration. Mortality data from these sources are combined to create a composite mortality variable for research purposes. Carelon Research has compared the composite mortality variable to the National Death Index and observed good agreement from 2010 to 2018 (sensitivity, 89%; positive predictive value, 93%).[3]

Data S2. Baseline clinical characteristics and clinical outcomes by first-line platinum-based chemotherapy (1L PBC) regimen.

Baseline clinical characteristics

- Among patients on 1L PBC, the proportion of patients with comorbidities was generally higher among those receiving carboplatin, and lowest among those on dose-dense methotrexate, vinblastine, doxorubicin, and cisplatin (DDMVAC).
- The proportion of patients with comorbidities for the cisplatin-based PBC, carboplatin-based PBC, and DDMVAC cohorts, respectively, was:
    - Chronic pulmonary disease (26.6%, 29.8%, and 16.4%)
    - Diabetes without chronic complications (19.5%, 29.7%, and 18.2%)
    - Renal disease (16.1%, 30.8%, and 9.1%)
    - Mild liver disease (24.1%, 20.7% and 25.5%)
    - Peripheral vascular disease (16.7%, 23.1%, and 16.4%).
- The proportion of patients with an Eastern Cooperative Oncology Group performance score of 0–1 was 95.9%, 90.1%, and 100% for cisplatin-based PBC, carboplatin-based PBC, and DDMVAC, respectively.

Clinical outcomes

- Median follow-up (interquartile range [IQR]) in months was 11.3 (5.8–20.3), 10.6 (5.1–20.0), and 15.3 (8.3–21.1) for patients on cisplatin-based PBC, carboplatin-based PBC, and DDMVAC.
- The overall survival probabilities at 6, 12, and 36 months were 90%, 78%, and 50% for cisplatin-based PBC; 84%, 68%, and 40% for carboplatin-based PBC; and 100%, 89%, and 62% for DDMVAC.
- The median (IQR) time on treatment was similar for cisplatin-based PBC (2.7 [1.4–3.4] months) and carboplatin-based PBC (2.7 [1.4–4.1] months), but shorter for DDMVAC (2.0 [1.4–2.0] months).
- The median time to next treatment among patients who initiated 2L treatment was similar with 1L cisplatin-based PBC (4.8 [2.8–7.6] months) and 1L carboplatin-based PBC (4.8 [2.8–9.0] months), but longer with DDMVAC (5.5 months [4.1–9.7]).
- The proportion of patients who died during follow-up was the highest in the carboplatin-based PBC cohort at 39.0%, compared with 26.9% in the cisplatin-based PBC cohort and 20.0% in the DDMVAC cohort.
    - The median (IQR) time from the date of the first urothelial carcinoma diagnosis to death in patients who received cisplatin-based PBC, carboplatin-based PBC, and DDMVAC was 13.5 (6.0–22.6), 12.8 (5.9–20.8), 17.6 (11.7–28.1) months, respectively.

Supplementary References

1. Price, A.; Chi, W.; Overhage, J.M. Implementation and validation of a prioritization logic to identify the best available race/ethnicity information for members in commercial plans. Poster presentation at the 2024 ISPOR Annual Meeting in Atlanta, Georgia, USA. Available online: https://www.ispor.org/heor-resources/presentations-database/presentation/intl2024-3898/139634 (accessed on 21 August 2024).
2. Kern, D.M.; Barron, J.J.; Wu, B.; Ganetsky, A.; Willey, V.J.; Quimbo, R.A.; Fisch, M.J.; Singer, J.; Nguyen, A.; Mamtani, R. A validation of clinical data captured from a novel Cancer Care Quality Program directly integrated with administrative claims data. *Pragmat. Obs. Res.* **2017**, *8*, 149-155.
3. Jamal-Allial, A.; Sponholtz, T.; Vojjala, S.K.; Paullin, M.; Papazian, A.; Eshete, B.; Mahmoudpour, S.H.; Verpillat, P.; Beachler, D. Evaluation of mortality data sources compared to the National Death Index in the Healthcare Integrated Research Database. *Pragmat. Obs. Res.* **2025**, *16*, 19–25.
